# Is there a relation between novelty seeking, striatal dopamine release and frontal cortical thickness?

**DOI:** 10.1371/journal.pone.0174219

**Published:** 2017-03-27

**Authors:** Natalia Jaworska, Sylvia M. Cox, Kevin F. Casey, Isabelle Boileau, Mariya Cherkasova, Kevin Larcher, Alain Dagher, Chawki Benkelfat, Marco Leyton

**Affiliations:** 1 Department of Psychiatry, McGill University, Montreal, Quebec, Canada; 2 Institue of Mental Health Research, Ottawa, Ontario, Canada; 3 Le Centre Hospitalier Universitaire (CHU) Sainte-Justine, Montreal, Quebec, Canada; 4 Centre for Addiction & Mental Health (CAMH), Toronto, Ontario, Canada; 5 University of British Columbia, Division of Neurology, Vancouver, British Columbia, Canada; 6 Montreal Neurological Institute (MNI), McGill University, Montreal, Quebec, Canada; Chiba Daigaku, JAPAN

## Abstract

**Background:**

Novelty-seeking (NS) and impulsive personality traits have been proposed to reflect an interplay between fronto-cortical and limbic systems, including the limbic striatum (LS). Although neuroimaging studies have provided some evidence for this, most are comprised of small samples and many report surprisingly large effects given the challenges of trying to relate a snapshot of brain function or structure to an entity as complex as personality. The current work tested *a priori* hypotheses about associations between striatal dopamine (DA) release, cortical thickness (CT), and NS in a large sample of healthy adults.

**Methods:**

Fifty-two healthy adults (45M/7F; age: 23.8±4.93) underwent two positron emission tomography scans with [^11^C]raclopride (specific for striatal DA D_2/3_ receptors) with or without amphetamine (0.3 mg/kg, p.o.). Structural magnetic resonance image scans were acquired, as were Tridimensional Personality Questionnaire data. Amphetamine-induced changes in [^11^C]raclopride binding potential values (ΔBP_ND_) were examined in the limbic, sensorimotor (SMS) and associative (AST) striatum. CT measures, adjusted for whole brain volume, were extracted from the dorsolateral sensorimotor and ventromedial/limbic cortices.

**Results:**

BP_ND_ values were lower in the amphetamine *vs*. no-drug sessions, with the largest effect in the LS. When comparing low *vs*. high LS ΔBP_ND_ groups (median split), higher NS2 (impulsiveness) scores were found in the high ΔBP_ND_ group. Partial correlations (age and gender as covariates) yielded a negative relation between ASTS ΔBP_ND_ and sensorimotor CT; trends for inverse associations existed between ΔBP_ND_ values in other striatal regions and frontal CT. In other words, the greater the amphetamine-induced striatal DA response, the thinner the frontal cortex.

**Conclusions:**

These data expand upon previously reported associations between striatal DA release in the LS and both NS related impulsiveness and CT in the largest sample reported to date. The findings add to the plausibility of these associations while suggesting that the effects are likely weaker than has been previously proposed.

## Introduction

Impulsive novelty seeking (NS) traits have been proposed to reflect individual differences in meso-striatal dopamine (DA) transmission [1],[2] and aspects of cortical morphometry, including grey matter volume and cortical thickness (CT) [3],[4]. The model is compelling. Limbic DA transmission potently influences the incentive salience of rewarding and potentially rewarding (novel) stimuli [5],[6],[7], and cortical projections densely innervate the striatum [8],[9] further modulating the planning of approach and avoidance behaviors [1],[10]. In humans, individual differences in CT, striatal DA D_2_ receptor availability and striatal DA responses to a drug challenge (e.g., amphetamine) have been reported to account for a substantial proportion of the variance in temperamental features such as NS (**Tables 1–3**). However, given the noise inherent to both neuroimaging data and personality trait measurements, it seems surprising that a single snapshot of a neurobiological feature can account for so much variation in temperament [11]. Since much of this work has been conducted in small samples, and rarely with the same measure of impulsivity, in the current study, we investigated—for the first time—the relation between NS, CT and amphetamine-induced striatal DA release (via regression analyses) in the largest sample of healthy adults reported to date. Based on the previous studies, we predicted, *a priori*, that greater DA release would be associated with higher impulsivity-related NS scores and thinner frontal cortices. Additionally, since the association between CT and DA release has only been previously reported once in a smaller cohort [12], we sought to replicate this finding in a substantially larger sample. The same is true for reproducing the association between striatal DA release and NS, which was reported only once previously [13].

**Table 1:** Associations between baseline meso-striatal dopamine function & externalizing personality traits.

Study	Participant Characteristics	Task/Questionnaire	Neuroimaging Measure	Findings	Interpretation
***Baseline striatal DA***_***R***_ ***availability (baseline BP***_***ND***_***) & externalizing personality traits***					
*Lee et al., 2009 [14]	N = 51methamphetamine-dependent adults(33.6±8.8, 21F/30M; PET N = 22) N = 66 (31.3±8.3, 32F/34M; PET N = 30) healthy adults	Questionnaire: BIS-11	[^18^F]fallypride (D_2/3_ DA_R_) -Relations between BIS & baseline BP_ND_ (controlling for age)	Negative correlation between baseline BP_ND_ in left caudate nucleus/right lateral putamen/claustrum & BIS (greater effect in dependent adults).	Low baseline striatal DA_R_ availability → higher impulsiveness.
Gjedde et al., 2009 [15]	N = 18 healthy M (30.1±7.1)	Questionnaire: Zuckerman scale (SS assessment)	[^11^C]raclopride (striatal D_2/3_ DA_R_) -Relations between SS & baseline BP_ND_	Inverted-U shape best fit BP_ND_ values in ventral striatum/putamen & entire range of SS.	Striatal baseline DA_R_ availability is lower in those with more or less SS than average.
Reeves et al., 2012 [16]	N = 23 healthy adults (47±11; 4F/19M)	Questionnaire: BIS-11	[^11^C]raclopride -Associations with personality & BP_ND_	After excluding those with high dissimulation scores: baseline limbic striatum BP_ND_ correlated positively with non-planning impulsivity (N = 14; *r* = .58).	Increased baseline striatal D_2/3_ DA_R_ → higher non-planning impulsivity.
*Boileau et al., 2013 [17]	N = 13 M with problematic gambling (32.5±8.5) N = 12 healthy M (33.8±11.2)	Slot machine game → not during imaging -Questionnaire: EPI	[^11^C]-(+)-PHNO (D_3_ DA_R_) & [^11^C]raclopride -Associations between BP_ND_ & personality, performance	In healthy M, baseline dorsal striatum [^11^C]raclopride BP_ND_ correlated inversely with impulsiveness & reinforcing effects of slot machine game (*r* = -.70).	Low baseline dorsal striatal DA_R_ availability → higher impulsiveness & gambling liking.
Kim et al., 2014 [18]	N = 21 healthy adults (34.6±8.8; 13F/8M)	Questionnaire: BIS-11 & TCI (HA & NS)	[^11^C]raclopride -Relations between NS & baseline BP_ND_ (HA & NS controlled for) -BP_ND_ comparisons between high *vs*. low BIS groups	Greater BP_ND_ in pre-commissural dorsal caudate in high *vs*. low BIS group. Non-planning/attentional impulsiveness correlated positively (*r* = .65/.61) with pre-commissural dorsal caudate BP_ND_.	Increased baseline DA_R_ availability in associative striatum → higher impulsiveness.
Robertson et al., 2015 [19]	N = 31 healthy adults (30.7±8.3; 16F/15M)	Tasks: SST & CPT (motor inhibition) → not during imaging	[^11^C]NNC-112 (D_1_ DA_R_) & [^18^F]fallypride -Correlations between performance & baseline BP_ND_ in striatum	SST RT negatively correlated with baseline striatal BP_ND_ (*r* = -.42 to -.62).	Low baseline striatal DA_R_ availability → more impulsive actions (which have been related to impulsivity traits)
*Oberlin et al., 2015 [20]	N = 10 alcoholics (33.3±6.8, 3F/7M) N = 13 (33.2±7.4, 6F/7M) healthy social drinkers	Monetary delay-discounting task (impulsive choice) → not during imaging -Questionnaire: I7	[^11^C]raclopride in LS-Correlations between performance & baseline BP_ND_	Greater impulsive choice for $20 correlated with lower right LS BP_ND_. Positive correlation between impulsiveness & left posterior putamen BP_ND_; between disinhibition & right anterior putamen BP_ND_.	Low baseline LS DA_R_ availability → greater impulsive choice. Increased baseline putamen DA_R_ availability → higher impulsive & disinhibitory traits.
***Summary*:** *Greater striatal DA*_*R*_ *availability may be associated with higher externalizing traits in healthy populations*, *though notable exceptions suggest the opposite or an inverted U-shaped association between DA*_*R*_ *availability and externalizing traits*.					
***Baseline striatal DA transporter availability*, *DA synthesis capacity & externalizing personality traits***					
Lawrence & Brooks, 2014 [21]	N = 12 healthy males (38±7)	Questionnaire: Disinhibition measured with TPQ NS2 (impulsivity) & NS3 (extravagance)	FDOPA (DA striatal synthesis capacity) -Correlations between disinhibition (controlling for HA) & baseline BP_ND_	Positive relation between NS3 & DA activity in LS (*r* = .78).	Greater DA synthesis capacity in LS → greater disinhibition (extravagance).
*Ishii et al., 2016 [22]	N = 16 patients with Parkinson’s Disease (66.3±7.2; 10F/6M) N = 28 healthy adults (10F/18M; 63.4±5.6; N = 17 in PET analysis)	Questionnaire: TCI (focus on NS & HA)	[^11^C]CFT (DA transporter availability) -Baseline BP_ND_ in striatum, limbic system & frontal lobe -DTI analysis	No correlations between NS/HA & baseline striatal [(^11^)C]CFT BP. NS correlated positively with connectivity between striatum-hippocampus/amygdala (both groups) & between striatum-DLPFC/left PFC (controls).	No relation between NS/HA & striatal DA transporter availability.
Smith et al., 2016 [23]	N = 16 healthy adults (28±2.7; 8F/8M)	Delay-discounting (DD) task (impulsive choice) → not during imaging	FMT (DA synthesis capacity) -Median split of baseline BP_ND_ in putamen & midbrain (VTA/SN)	Lower putamen FMT predicted greater ‘Now’ bias, more rapidly declining discount rate with increased delay & reduced willingness to accept better delayed rewards. Lower midbrain FMT predicted greater sensitivity to increasing magnitude of ‘Later’ reward.	Lower baseline putamen DA synthesis → greater impulsivity. Lower baseline midbrain DA synthesis → less impulsivity.
***Summary*:** *Inconclusive evidence for an association between DA transporter availability as well as DA synthesis capacity and externalizing traits in healthy populations*.					
***Baseline midbrain autoreceptor DA***_***R***_ ***availability & externalizing personality traits***					
Zald et al., 2008 [24]	N = 34 healthy adults (23.4; 16F/18M)	Questionnaire: TPQ (NS)	[^18^F]fallypride -SN/VTA *baseline BP*_*ND*_ -Relations between NS & baseline BP_ND_	Inverse correlation between NS & DA_R_ in SN/VTA (*r* = -.44).	Decreased baseline midbrain DA_R_ availability (autoreceptor) → increased NS.
*Savage et al., 2014 [25]	N = 8 normal weight females (38) N = 19 obese females (38)	Questionnaire: TPQ (NS)	[^18^F]fallypride -*Baseline BP*_*ND*_* *in SN -Correlations between NS & baseline BP_ND_	In normal weight group: negative correlation between NS & baseline SN D_2/3_ DA_R_ BP_ND_ (*r* = -.70).	Decreased baseline midbrain DA_R_ availability (autoreceptor) → increased NS.
***Summary*:** *Emerging evidence for an inverse association between midbrain autoreceptor DA*_*R*_ *availability and externalizing traits in healthy populations*.					

*Focus on healthy-controls or findings across all groups; Means ± standard deviations presented. CFT: 2β-carbomethoxy-3β-(4-fluorophenyl)-[N-^11^C-methyl]tropane; CPT: Continuous performance task; BIS-11: Barratt inhibition scale; BP_ND_: Binding potential, non-dissociable; D_2/3_ DA_R_: Dopamine (DA) receptors–subtypes 2 & 3; DTI: Diffusion tensor imaging; HA: Harm-avoidance; EPI: Eysenck Personality Inventory; FDOPA: 6-[^18^F]Fluoro-L-DOPA; FMT: 6-[^18^F]fluoro-l-m-tyrosine; I7: Impulsiveness-Venturesomeness-Empathy questionnaire; LS: Limbic striatum; M/F: Male/female; NS: Novelty-seeking; PHNO: [^11^C]-(+)-propyl-hexahydro-naphtho-oxazin; PET: Positron emission tomography; SN: Substantia nigra; SS: Sensation-seeking; SST: Stop-signal task; TCI: Temperament & Character Inventory; TPQ: *Tridimensional Personality Questionnaire*; VTA: Ventral tegmental area

**Table 2:** Associations between amphetamine-induced striatal dopamine release & externalizing personality traits.

Study	Participant Characteristics	Task/Questionnaire	Neuroimaging Measure	Findings	Interpretation
Leyton et al., 2002 [13]	N = 8 healthy M (29.9±8.7)	*d*-amphetamine challenge (.3 mg/kg p.o.) & placebo -Questionnaire: TPQ	[^11^C]raclopride (striatal D_2/3_ DA_R_) -ΔBP_ND_ (no-drug *vs*. drug) -Associations between TPQ & ΔBP_ND_	Amphetamine decreased LS BP_ND_. ΔBP_ND_ correlated positively with NS & NS-exploratory excitability (*r* = .56 & .55).	Greater drug-induced striatal DA responses → higher NS.
Riccardi et al., 2006 [26]	N = 14 healthy adults (25.9; 6F/7M)	*d*-amphetamine challenge (.43 mg/kg, p.o.) -Questionnaire: SS Scale Form V	[^18^F]fallypride (D_2/3_ DA_R_) -ΔBP_ND_ (no-drug *vs*. drug) -Associations between SS & ΔBP_ND_	SS correlated positively with DA response in left LS in M (*r* = .90); opposite in F (*r* = -.71). F: negative correlation between SS & DA response in caudate head, globus pallidus & left anterior cingulate. M: negative correlation between SS & DA response in insula, inferior temporal cortex & left lateral thalamus.	Differential relations between DA activity depending on region & sex.
Oswald et al., 2007 [27]	N = 20 healthy adults—high impulsivity (21.6±3.3; 9F/11M) N = 20—low impulsivity (22.4±3.0; 5F/15M)	*d*-amphetamine challenge (.3 mg/kg i.v.) -Questionnaire: NEO-PI—high/low impulsivity groups	[^11^C]raclopride -ΔBP_ND_ (no-drug *vs*. drug) -High *vs*. low impulsivity comparisons	High *vs*. low impulsivity subjects had lower right LS DA under conditions of low/moderate stress.	High trait impulsivity → blunted right LS DA release (moderated by stress).
Buckholtz et al., 2010 [28]	N = 32 healthy adults (22.6; 16F/16M)	*d*-amphetamine challenge (.43 mg/kg oral) & placebo -Questionnaire: BIS-11	[^18^F]fallypride -ΔBP_ND_ (no-drug *vs*. drug) in SN/VTA & striatum -Associations between BIS & ΔBP_ND_	BIS-11 predicted midbrain baseline DA_R_ BP_ND_ (greater scores, fewer receptors). Greater BIS-11 scores → greater LS DA release. SN/VTA DA_R_ BP_ND_ → inversely correlated with striatal DA release. Increased striatal DA release predicted drug wanting (right/left: *r* = .47 & .48).	Greater striatal DA response → greater impulsiveness. Lower midbrain DA autoreceptor availability may lead to impulsivity by enhancing striatal DA release (mediation).
*Cherkasova et al., 2014 [29]	N = 15 M with ADHD (29.9±8.7) N = 18 healthy M (25.4±6.8)	*d*-amphetamine challenge (.3 mg/kg p.o.) -Task: SST	[^11^C]raclopride -ΔBP_ND_ (no-drug *vs*. drug) -Associations between performance & ΔBP_ND_	Entire group: larger striatal ΔBP_ND_ decreases (AST/SMST) → higher inattention scores (*r* = .47 & .43). RTs tended to positively correlate with AST/SMST ΔBP_ND_.	Greater striatal DA response → greater inattention & poorer response inhibition.
Oswald et al., 2015 [30]	N = 45 healthy adults (22.7±3.0; 18F/27M)	*d*-amphetamine challenge (.3 mg/kg i.v.) -Iowa Gambling Task → not during imaging	[^11^C]raclopride -ΔBP_ND_ (no-drug *vs*. drug) -Associations between performance & ΔBP_ND_	Riskier decisions → greater right LS DA release (adjusted for stress & sex).	Greater LS DA release → riskier decision making.
***Summary*:** *Most evidence suggests that higher externalizing traits and behaviors in healthy individuals are associated with an increased striatal DA response to an amphetamine challenge*.					

*Focus on healthy-controls or findings across all groups; Means ± standard deviations presented. ADHD: Attention deficit hyperactivity disorder; AST: Associative striatum; BIS-11: Barratt Inhibition Scale; BP_ND_: Binding potential, non-dissociable; D_2/3_ DA_R_: Dopamine receptors–dopamine 2 & 3 receptor subtypes; LS: Limbic striatum; M/F: Male/female; NEO-PI: Neuroticism, Extroversion, Openness Personality Inventory; NS: Novelty-seeking; SMST: Sensorimotor striatum; SN: Substantia nigra; SS: Sensation-seeking; SST: Stop-signal task; TPQ: *Tridimensional Personality Questionnaire*; VTA: Ventral tegmental area

**Table 3:** Associations between frontal cortical thickness & externalizing personality traits.

Study	Participant Characteristics	Task/Questionnaire	Neuroimaging Measure	Findings	Interpretation
Schilling et al., 2012 [31]	N = 32 healthy adults (35.2±10.5; 18F/14M)	Questionnaire: BIS-11	Associations between CT & personality measures	Negative correlation between left middle frontal gyrus CT & BIS. OFC/superior frontal gyrus CT inversely correlated with BIS.	Thinner frontal cortices → greater impulsiveness.
Schilling et al., 2013 [3]	N = 1620 healthy youth (14.43±0.39; 866F/754M)	Questionnaire: TCI	Associations between CT & personality measures	Inverse correlation between impulsiveness & left superior frontal CT.	Thinner frontal cortices → greater impulsiveness.
*Jiang et al., 2015 [32]	N = 30 healthy youth (15.1±0.6; 9F/21M) N = 28 youth with conduct disorder (14.8±0.8; 6F/22M)	Questionnaire: BIS-11	Associations between CT & personality measures	All participants: negative correlation between BIS & lateral OFC (*r* = -.43, corrected for multiple comparisons).	Thinner OFC cortices → greater impulsivity in adolescents.
Holmes et al., 2016 [4]	N = 1234 healthy adults (18–35)	Questionnaire: BIS-11 SS composite score: TCI-NS & fun-seeking (BIS/BAS) & risk-taking (Domain-Specific Risk-Attitude Scale)	Associations between CT & personality measures	Increased composite score SS & motor impulsivity were associated with reduced CT in regions implicated in cognitive control (ACC & middle frontal gyrus; *r* = -.11 to -.15).	Thinner CT in cognitive control circuitry → greater SS & motor impulsivity.
***Summary*:** *Most evidence suggests that higher externalizing traits are associated with thinner frontal cortices in healthy individuals*.					

*Focus on healthy-controls or findings across all groups; Means ± standard deviations presented. BIS/BAS: Behavioral Inhibition & Behavioral Activation Scales; BIS-11: Barratt Inhibition Scale; CT: Cortical thickness; M/F: Male/female; NS: Novelty-seeking; OFC: Orbitofrontal cortex; SS: Sensation-seeking; TCI: Temperament & Character Inventory

## Materials and methods

### Overview

Data were pooled from five previously reported studies [12],[13],[29],[33],[34], involving healthy adults (N = 52) who underwent two positron emission tomography (PET) [^11^C]raclopride scans, with and without amphetamine. Structural magnetic resonance image (MRI) scans and Tridimensional Personality Questionnaire (TPQ) data were also acquired [35], with a focus on the relation between neuroimaging variables and NS, including NS_Total_ scores and the constituent subscales of exploratory excitability (NS1), impulsiveness (NS2), extravagance (NS3) and disorderliness (NS4).

### Participants

Participants were healthy men (n = 45) and women (n = 7). Following a telephone screen, volunteers underwent an in-person physical exam, standard laboratory tests and a psychiatric assessment (Structured Clinical Interview for DSM-IV-TR [SCID-IV-TR], non-patient edition [36]). Inclusion criteria were as follows: a) absence of axis I disorder, b) no first-degree relative with a substance use disorder, c) no major medical conditions, d) no current/past neurological issues (e.g., no loss of consciousness for >5 min), e) negative seropositive pregnancy test in females. Volunteers with modest alcohol and infrequent recreational drug use (e.g., hallucinogens, marijuana) were not excluded (see individual papers for further details). All participants provided written informed consent prior to starting the studies, which were carried out in accordance with the Declaration of Helsinki and approved by the Research Ethics Committee of the Montreal Neurological Institute (MNI), McGill University.

### Neuroimaging acquisition

PET scans were carried out on two separate days (inter-scan time: 23.6 days±26.9; range: 1–104 days). Participants were asked to abstain for a minimum of 7 h and 24 h from tobacco and alcohol, respectively. Preliminary evidence suggests that there is not an effect of menstrual phase on [^11^C]raclopride BP_ND_ values [37], but females were tested during the follicular phase of their cycle. All participants tested negative on a urine drug screen (Triage Drugs of Abuse Panel, Biosite Diagnostics) prior to each scan.

PET scans were acquired, with and without ingesting a capsule filled with amphetamine (0.3 mg/kg, p.o.) 60 min prior to tracer injection, with a Siemens ECAT HR+ PET scanner (CTI/Siemens, Knoxville, TN, USA; 63 slice coverage; maximum resolution of 4.2 mm; full-width half-maximum [FWHM] in the center of the field of view). In three of the studies, a placebo capsule was administered, while in two studies a baseline scan was obtained (i.e., no placebo capsule; study was used as a covariate, when appropriate, and details are provided in the following sections). Immediately after the transmission scan (for attenuation correction), [^11^C]raclopride (8–10 mCi) was injected as a bolus into the antecubital vein. Emission data were collected over 60 min in 26 time frames of progressively longer durations.

For anatomical co-registration with PET scans and CT analysis, T1-weighted MRIs were acquired with a 1.5 T Siemens scanner (1 mm slice thickness; TR = 9.7 ms, TE = 4 ms, flip angle = 12°, 256×256 matrix or TR = 22 ms, TE = 9.2 ms, flip angle = 30°, 256 × 256 matrix).

### PET analysis

#### Region of interest (ROI) analysis

The striatum was divided into 6 functional ROIs, defined on each participant’s MRI (automated segmentation using in-house Automatic Nonlinear Image Matching and Anatomical Labeling [ANIMAL]; ROI-MRI overlap was visually inspected). ROIs were the left/right sensorimotor striatum (SMS; post-commissural putamen), associative striatum (ASTS; pre-commissural dorsal caudate and dorsal putamen, post-commissural caudate) and ventral limbic striatum (LS) [38]. Parametric images were generated by deriving [^11^C]raclopride BP_ND_ values from each ROI using a simplified kinetic model that uses the cerebellum as a reference tissue devoid of DA D_2/3_ receptors to describe the kinetics of the free and specifically bound ligand [39]. Mean BP_ND_ values were extracted from each ROI in the conditions with and without amphetamine. During rest, BP_ND_ is proportional to the concentration of available D_2/3_ receptors; during stimulation, decreases in BP_ND_ are proportional to increases in extracellular DA. Amphetamine-induced change in BP_ND_ (ΔBP_ND_) was calculated as follows: [(placebo-amphetamine)/placebo]×(100)]. The greater the positive ΔBP_ND_ values, the greater the amphetamine-induced DA release [40].

#### Voxel-wise analysis

PET images were co-registered to MRIs and parametric [^11^C]raclopride BP_ND_ data were generated at each voxel using a simplified reference tissue model (cerebellum). Images were normalized into standardized (MNI) space. An 8 mm Gaussian smoothing kernel (FWHM) was applied to PET images (2 individuals excluded due to co-registration problems between BP_ND_ images/conditions; N = 47). A t-map was created to assess changes in [^11^C]raclopride BP_ND_ between scans with and without amphetamine using a residual t-statistic (BP_ND_ baseline>BP_ND_ amphetamine) [41]. In this approach, residuals are used to calculate the variance in parameter estimates; this method has been used extensively and validated using Monte Carlo simulations and real PET data [41],[42]. Significant voxels were identified by thresholding the t-map at t>4.1 (*p*<.05, Bonferroni corrected for multiple comparisons, search volume of the entire striatum).

### Cortical thickness (CT) analysis

MRIs were processed using the CIVET 2.0.0 pipeline with CBRAIN (https://cbrain.mcgill.ca/). The pipeline involves: a) Normalizing each image to standardized space (MNI ICBM-152 template). b) Correction of intensity non-uniformity artifacts. c) Tissue type classification. d) Fitting images with a deformable mesh model to extract 2D inner (white/gray matter interface) and outer (pial) cortical surfaces using the CLASP algorithm [43]. This generates CT measurements at 40,962 vertices/hemisphere (distance between outer CSF/gray matter and gray/white matter interfaces). e) Surfaces are registered to the MNI ICBM-152 surface template. f) Reverse linear transformations allow CT estimations in native space. g) CT is calculated at each cortical point using the t_link_ metric [44]. h) Smoothing with a 20 mm FWHM kernel.

Subsequently, CT vertex values were averaged into 74 regions (37/hemisphere) using the AAL label set [45] [analyzed using SurfStat (http://www.math.mcgill.ca/keith/surfstat/), a toolbox that runs on MATLAB (The MathWorks, Inc., MA, USA; http://www.modelgui.org/mgui-neuro-civet-matlab)]. Further reduction of frontal CT regions was carried out based on precedent connectivity analyses between the striatum and fronto-cortical regions. Specifically, three frontal clusters were created: a) dorsolateral prefrontal cortex (DLPFC: superior frontal gyri, dorsolateral; middle frontal gyri; inferior frontal gyri—triangular part; inferior frontal gyri—opercular part), b) sensorimotor cortex (SM: supplementary motor area; paracentral lobule; pre- & post-central gyri) and c) ventromedial PFC (vmPFC) and limbic cortex (vmPFC/limbic: olfactory cortex; gyrus rectus; middle, superior & inferior frontal gyri—orbital part; anterior cingulate; paracingulate gyri), based on structural and functional connections with the ASTS, SMS and LS, respectively [46]. CT measures were adjusted by total brain volume (regional CT/total brain volume×10^6^, as in Zhou et al., 2014); all CT statistics reflect adjusted CT [47].

### Statistics

Unless stated otherwise, means, standard deviations (SD) and partial eta-squares (*η*^2^_*partial*_: effect sizes) are presented. To characterize the effect of amphetamine-induced DA release, repeated-measures analysis of covariance (rmANOCVA; study as covariate since preliminary analyses revealed a main effect of study on BP values) was carried out on BP_ND_ values with drug condition (no amphetamine, amphetamine) and striatal ROI (LS, ASTS, SMS) as within-subject factors. Since no hemisphere effects were seen in preliminary analyses, it was not included as a factor. rmANOVAs were carried out on ΔBP_ND_ values, with striatal ROI as the within-subject factor (study was not used as a between-subject factor since preliminary analyses yielded no main effect of study on ΔBP_ND_ values). Finally, we carried out a median split of ΔBP_ND_ in the LS, yielding high *vs*. low amphetamine responders, and univariate ANCOVAs tested for group (high *vs*. low responders) differences in NS scores (age as covariate). Since NS and other impulsivity traits generally decrease as adults grow older, age was used as a covariate [48] (although our sample was relatively young, we noted an inverse correlation between NS3 and age: *r* = -.33, N = 52, *p* = .017).

Partial correlations were carried out between BP_ND_ values per drug condition in each striatal ROI (6 total) and NS scores (5 total: 4 subscales and NS_Total_; age and study controlled for), only *p*<.005 results were reported (i.e., .05/11 = .0045). Partial correlations (age controlled for) were also carried out between ΔBP_ND_ in each striatal ROI (3 total) and NS scores (5 total); only *p*<.006 results were reported (i.e., .05/8 = .0063).

rmANCOVAs were carried out on CT measures with frontal region (DLPFC, SM, vmPFC/limbic) as the within-subject factor; age and gender were used as covariates [49]; preliminary analyses indicated no hemispheric effects, thus, it was not included as a factor). Study was not used as a factor as preliminary analyses yielded no effect of study on CT. Partial correlations (controlling for age and gender) were carried out between CT measures in the frontal regions (averaged across hemispheres) and NS scores (5 total); only correlations with *p*<.006 were reported (i.e., .05/8 = .006).

Partial correlations (controlling for age, gender and study) were also carried out between frontal CT values (3 regions) and BP_ND_ measures (3 ROIs) in each drug condition (amphetamine, baseline/placebo); correlations between CT values and ΔBP_ND_ were also carried out (controlling for age and gender). Significance was set at *p* = .006 (i.e., .05/8) and *p* = .008 (i.e., .05/6), respectively.

Finally, we carried out stepwise multiple regressions to assess the influence of CT measures (3 regions) and NS scores (5 total) on ΔBP_ND_ in each striatal ROI. Age and gender were also included as predictor variables (stepwise). Multicollinearity was assessed using the variance inflation factor (VIF; <1.5 deemed acceptable); autocorrelations in the residuals were assessed using the Durbin Watson statistics (acceptable: 1.5-2.5). The significance of the ANOVA was *p*<.005 (i.e., *p*<.05/11 factors). Unstandardized beta coefficients are presented.

## Results

### Participant characteristics

Detailed participant characteristics can be found elsewhere [12],[13],[29],[33],[34]. CT data were available from all 52 participants (18–42 yr, **Table 4**); BP_ND_/ΔBP_ND_ data were available from 49 participants (3 PET scans could not be used because of image quality/technical issues).

**Table 4:** Participant Characteristics.

Characteristics	Mean (SD)
Sex	7F/45M
Age	23.77 (4.93)
NS1 (exploratory excitability)	6.44 (1.44)
NS2 (impulsiveness)	3.12 (1.85)
NS3 (extravagance)	4.27 (1.56)
NS4 (disorderliness)	5.69 (1.77)
NS Total	19.52 (4.52)

NS: Novelty seeking; F: Females; M: Males

### BP_ND_ & ΔBP_ND_ results

**Fig 1:**
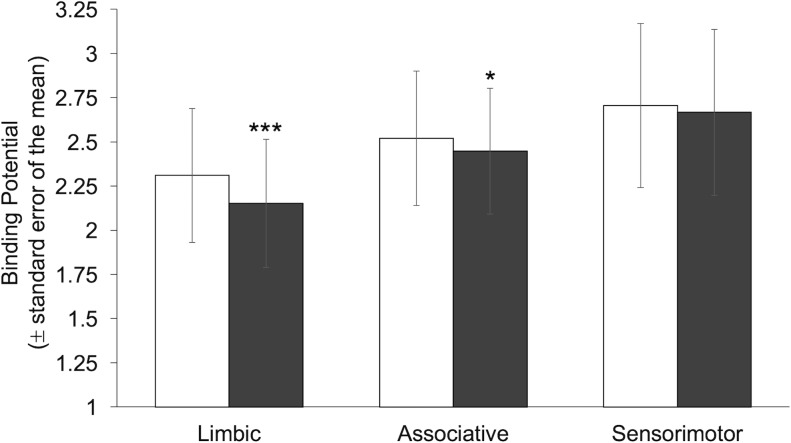
Striatal binding potentials. Mean binding potentials (BP_ND_ ± standard error of the mean) in the limbic, associative and sensorimotor striatum with (grey) and without (white) amphetamine (***p<.001, *p<.05).

No sphericity violations existed, and there was no relation between inter-scan time duration (days) and ΔBP_ND_. A main effect of study existed for BP_ND_ [F(1,44) = 3.55, *p* = .04, *η*^2^_*partial*_ = .24]; study was used as a covariate in BP_ND_ value analyses. The rmANCOVA of BP_ND_ values yielded main effects of drug condition [F(1,44) = 6.44, *p* = .02], reflecting lower BP_ND_ values on the sessions with *vs*. without amphetamine (2.45±.40 *vs*. 2.55±.41, *η*^2^_*partial*_ = .13), and ROI [F(2,88) = 55.29, *p*<.001, *η*^2^_*partial*_ = .56], reflecting a gradation of BP_ND_ values from LS to ASTS [*p*s<.001; LS: 2.25±.40, ASTS: 2.52±.40, SMS: 2.73±.51]. In this case, lower BP_ND_ values indicated greater extracellular DA in the synapse (i.e., inverse relation between BP_ND_ values and DA). A drug condition×ROI interaction was also evident [F(2,88) = 3.73, *p* = .028, *η*^2^_*partial*_ = .08], reflecting significant effects of amphetamine in the LS (*p*<.001, placebo: 2.33±.38, amphetamine: 2.17±.36) and ASTS (*p* = .045; placebo: 2.56±.38, amphetamine: 2.48±.36) but not SMS (placebo: 2.71±.46, amphetamine: 2.67±.47; **Fig 1**).

**Fig 2:**
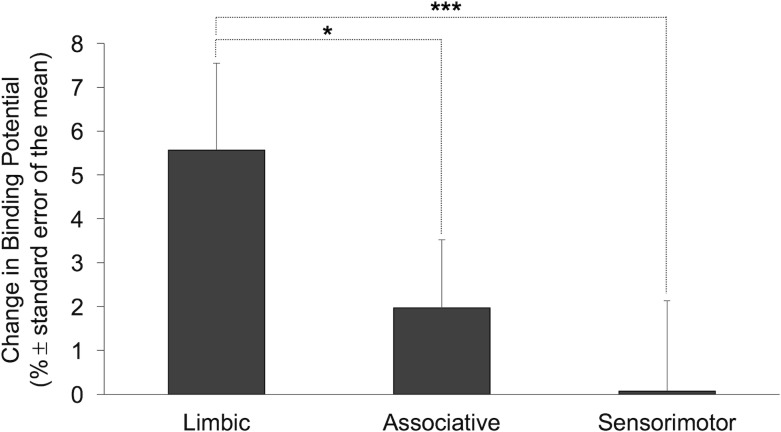
Amphetamine-induced changes in binding potentials. Amphetamine-induced changes (%) in binding potentials (ΔBP_ND_ ± standard error of the mean) in the limbic, associative and sensorimotor striatum. More positive ΔBP_ND_ values reflect greater amphetamine-induced dopamine release (*p<.05 and ***p = .001, respectively).

**Fig 3:**
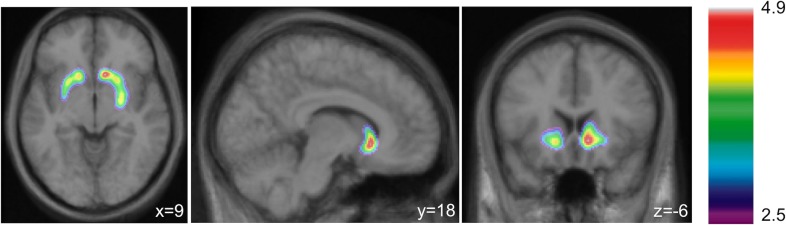
Voxel-wise t-map of binding potential. Voxel-wise t-map of [^11^C]raclopride binding potential (BP_ND_) illustrating reduced BP_ND_ (i.e., greater dopamine release) following amphetamine *vs*. baseline (*t*>4.1, *p*<.05 corrected). No significant voxels were noted outside the striatum.

The rmANOVA assessing ΔBP_ND_ values yielded a main effect of ROI [F(2,96) = 7.84, *p* = .001, *η*^2^_*partial*_ = .14, study was not used as a covariate as there was no study effect on ΔBP_ND_]. Follow-up comparisons indicated that ΔBP_ND_ values in the LS were larger than those in the ASTS (*p* = .017) and SMS (*p* = .001; **Fig 2**). Voxel-wise analysis confirmed reduced BP_ND_ following amphetamine administration in four clusters, with the greatest effect in the right LS (**Table 5; Fig 3**).

**Table 5:** Voxel-wise analysis of [^11^C]raclopride binding potential (BP_ND_) difference values between placebo/baseline and amphetamine.

Region	Peak coordinates (MNI: x, y, z)	Peak T-value (*p* value)	Cluster size–voxels (mm^3^; *p* value)
**Ventral Striatum (LS)**			
Right	9, 18, -6	4.91 (*p =* .003)	67 (536; *p*
Left	-15, 15, -7	4.4	16 (124; *p* = .031)
**Putamen**			
Left anterior putamen (ASTS)	-29, 1, -2–25, 12, -3	4.53 (*p =* .017) 4.49 (*p =* .019)	48 (387; *p* = .001)
Left posterior putamen (SMS)	-30, -2, -2	4.5 (p = 0.019)	
Right posterior putamen (SMS)	25, -5, -4	4.4 (*p =* .028)	20 (163; *p* = .016)

Corrected *p* values presented. LS: Limbic striatum; ASTS: Associative striatum; SMS: Sensorimotor striatum

**Fig 4:**
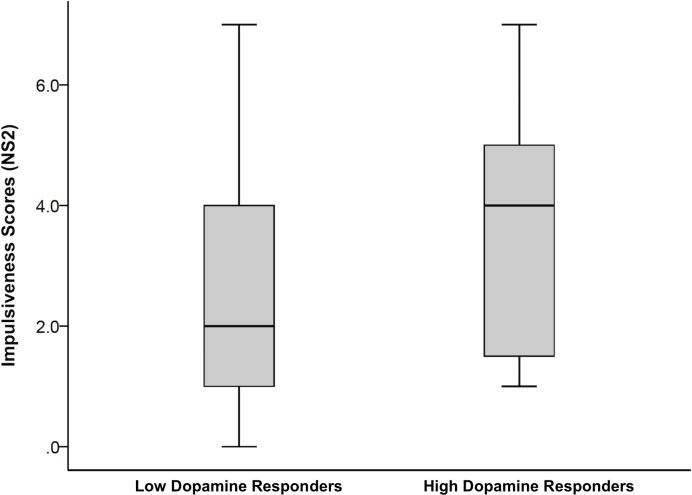
Impulsiveness scores in high and low amphetamine responders. Boxplots of impulsiveness scores (NS2) in high and low dopamine (DA) responders reflecting the extent of binding potential changes (ΔBP_ND_) in the limbic striatum (LS) to amphetamine (based on a median-split).

The univariate ANCOVA (age as a covariate) yielded a trend for a difference between the low *vs*. high LS ΔBP_ND_ groups on NS2 (impulsiveness) [F(1,46) = 3.20, *p* = .07, *η*^2^_*partial*_ = .07; based on our *a priori* hypothesis: *p* = .035], with higher scores in the high LS ΔBP_ND_ group (3.63±1.88; low: 2.68±1.80; **Fig 4**).

### Correlations between BP_ND_/ΔBP_ND_ & NS scores

No significant partial correlations (significance was set at *p*<.005/006) existed between NS scores and ΔBP_ND_ values.

### CT measures & correlations with NS scores

A rmANCOVA (age and gender as covariates) yielded a main effect of region [F(2,98) = 4.05, *p* = .02, *η*^2^_*partial*_ = .08], with cortical thickness greatest in the limbic region and thinnest in the SM region (*p*s<.001). Study was not used as a covariate since preliminary analyses yielded no main effect of study on CT. No significant partial correlations between CT and NS scores emerged (gender and age were used as covariates, significance set at *p*<.006). When age and gender were not controlled for, exploratory Spearman’s correlations indicated that there was an inverse correlation between thickness in the vmPFC/limbic cortex and NS3 scores (extravagance; *rho* = -.38, *p* = .006, N = 52).

### Correlations between CT & BP_ND_/ΔBP_ND_

**Fig 5:**
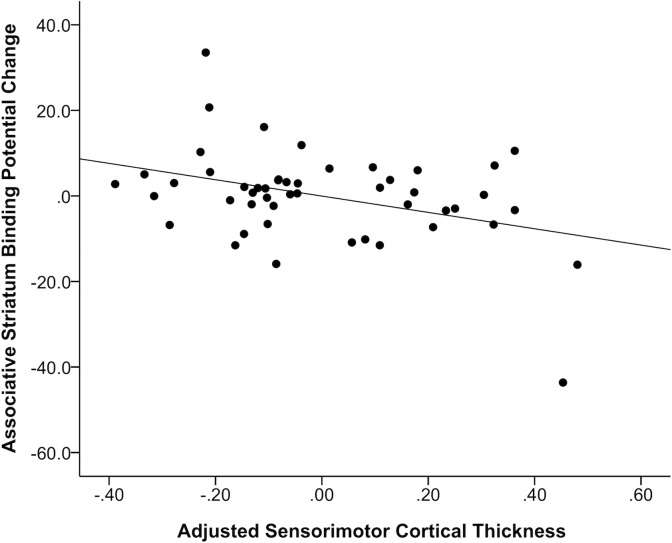
Partial correlations between associative striatal dopamine release and sensorimotor cortical thickness, controlling for age and gender. Correlation between the residuals from regressing change in binding potentials (% ΔBP_ND_) in the associative striatum on age and gender, and the residuals from regressing adjusted sensorimotor cortical thickness on age and gender.

Partial correlations (age and gender controlled for) yielded a negative relation between ASTS ΔBP_ND_ and CT in the SM region (*r* = -.38, df = 45, *p* = .008; **Fig 5**); a trend for an inverse relation also existed between LS ΔBP_ND_ and CT in the SM region (*r* = -.36, *p* = .013) and between ASTS ΔBP_ND_ and CT in the DLPFC (*r* = -.35, *p* = .015). For each association, the thinner the cortex, the greater the amphetamine-induced DA response. No significant associations between baseline BP_ND_ values and CT were observed (age, gender and study controlled for).

### Multiple regressions

No autocorrelation or multicollinearity violations were noted. Stepwise multiple regression analysis yielded no regressions at the *p*<.005 level. LS ΔBP_ND_ was weakly associated with CT in the SM cortex (F[1,37] = 5.88, *p* = .019; R^2^ = .11, adjusted R^2^ = .09; predicted LS ΔBP_ND_ = 56.84+[-20.70][SM CT]). Similarly, ASTS ΔBP_ND_ scores were modestly associated with CT in the SM cortex (F[1,47] = 7.21, *p* = .01; R^2^ = .13, adjusted R^2^ = .12; predicted SMS ΔBP_ND_ = 46.22+[-17.86][SM CT]).

## Discussion

In the current study, we examined the relation between NS, frontal CT and striatal DA responses to an amphetamine challenge in a large sample of young healthy adults. The primary findings were that the greatest amphetamine-induced changes in BP_ND_ occurred in the ventral limbic striatum (LS). Individuals with high *vs*. low LS ΔBP_ND_ responses (high *vs*. low drug responders) had higher impulsiveness (NS2) scores. Partial correlations indicated that greater amphetamine-induced striatal DA responses are associated with thinner frontal cortices.

As expected, BP_ND_ values decreased following amphetamine administration. Thanks to the greater statistical power afforded by the large sample size, this study was able to convincingly demonstrate regional differences in the magnitude of this effect, with larger ΔBP_ND_ responses in the LS *vs*. other striatal regions, consistent with smaller PET studies in humans [13],[34],[46], [50],[51] and microdialysis studies in laboratory animals [52]. Via our voxel-wise analysis, we also confirmed that, with the *d*-amphetamine dose and route of administration used, there are no statistically significant changes in [^11^C]raclopride binding in voxels outside the striatum.

The mechanism accounting for the different magnitude of DA responses within striatal sub-regions requires more study, but limbic striatal inputs from the ventral tegmental area (VTA) and dorsal substantial nigra (SN) express fewer D_2_ autoreceptors and DA transporters (DAT) than projections to other striatal regions [53]. Decreased D_2_ autoreceptor expression may be associated with greater amphetamine-induced DA release in the LS (*vs*. other striatal regions) [28],[54], though the effect of fewer DATs is less clear [38],[55]. The LS also sends non-reciprocal GABAergic projections to the ASTS, as such, ASTS activity is regulated by LS GABAergic input (similarly, the ASTS appears to regulate the SMS via GABA projections) [38],[55]. Finally, the LS has extensive connections with the orbitofrontal cortex (OFC), vmPFC, and aspects of the anterior cingulate cortex (ACC) [46] as well as the amygdala and hippocampus [55]. Together, these systems play a pivotal role in the initiation and inhibition of approach toward rewarding and potentially rewarding stimuli [56].

The ΔBP_ND_ values in the current study are smaller than what has been reported previously with smaller samples [12],[13],[29],[33],[34]. This likely reflects that, in the current study, we used an automated PET analysis approach (i.e., an in-house PET analysis pipeline, involving few manual data corrections specifically with respect to co-registration between structural MRI and BP images; the pipeline employs an iterative co-registration process) that integrates Turku PET centre tools (http://www.turkupetcentre.net/)) for ROI analysis, which relies on traditional non-linear fitting to estimate Simplified Reference Tissue Model [SRTM] parameters. This approach is different from those used by the individual studies, which calculated SRTM parameters for ROI analysis using a basis function [39]. Perhaps most importantly, the automated pipeline employed ROIs that were larger than those used in some of the other studies. As a result, the correction for multiple comparisons was greater. Finally, we applied double-erosion (2 voxels), which may have excluded peak activation in the inferior and ventral-most aspects of the LS. These methodological differences may have influenced our BP and ΔBP_ND_ ROI values. However, given the large sample and different methods used previously, the automated pipeline improved the objectivity of the analyses, and the use of more stringent significance detection approaches in the ROI analyses minimized false positives.

Consistent with our *a priori* hypothesis [28],[13],[30] high *vs*. low amphetamine responders (i.e., those with greater DA release to amphetamine) exhibited higher NS2 (impulsiveness) scores. That this effect was subtle should not be surprising. There is inherent variability in neuroimaging indices, and a snapshot of a single aspect of function in a particular neural system (or morphometry) is unlikely to capture even an unvarying trait with minimal noise [11]. Measures of externalizing traits suffer from the same limitations. Moreover, any one measure of personality is unlikely to encapsulate the aspect most closely related to the neuroimaging metric being assessed; indeed, different aspects of externalizing traits may reflect various aspects of meso-striatal DA system function [57]. Nevertheless, the consistency with which measures of striatal DA transmission have been found to be associated with impulsive/externalizing traits (**Table 2**), along with the current study, suggests that individuals with higher impulsiveness scores are characterized by a more pronounced DA response to an amphetamine challenge. Based on the extensive connections with cortical regions implicated in emotion regulation [46], greater LS DA responses may facilitate more disinhibitory, impulsive-like behaviours to novelty and rewards.

The most novel aspect of this study was our attempt to relate striatal DA activity, frontal CT and NS traits in a large, healthy population. Inconsistent with our hypotheses, however, regression analyses indicated that the combination of NS and CT did not yield a stronger statistical prediction of striatal DA responses, as compared to either variable alone (i.e., although CT was associated with ΔBP_ND_, inclusion of NS did not improve the model). We found that greater DA responses in the ASTS were correlated with thinner sensorimotor cortices; similar tendencies existed between LS DA response and sensorimotor CT as well as between ASTS DA response and DLPFC CT. This is consistent with previous work by our group in a smaller sample, which was included in the current study [12]. To our knowledge, no other published data exist regarding the relation between striatal DA activity and cortical morphometry in healthy individuals. However, disease states characterized by striatal DA dysfunction, including Parkinson’s disease [58],[59], schizophrenia [60] and substance use disorders [61],[62], are typically associated with widespread cortical thinning as well as volume loss of the grey matter. Although brain morphometric changes in such disorders can be accounted for by numerous factors, and are certainly not solely related to striatal DA activity modifications, these findings suggest, at least to a certain extent, a relation between cortex morphometry and striatal DA activity.

Previous work found that OFC metabolism was inversely associated with drug-induced striatal DA release in controls [63], consistent with the idea that the OFC plays an integral role in reward valuation by way of LS activity regulation. Additionally, thicker PFCs are generally associated with improved cognition [64]. As such, a thicker PFC may index functional integrity, including more effective engagement of regulatory processes in the presence of rewards and other salient cues. Given the significant overlap between cortico-striatal loops, it is perhaps not surprising that we did not see specific correlations between ΔBP_ND_ in distinct striatal regions (ASTS, LS, SMS) and CT in corresponding fronto-cortical regions.

### Conclusion & limitations

This is the first known study to simultaneously assess *in vivo* striatal DA release in relation to CT and NS in humans. This noted, the results should be considered in light of the following. First, the no drug condition contained a placebo in some studies but not all. However, there were no differences in ΔBP_ND_ between studies with *vs*. without a placebo capsule, and study was used as a covariate in the statistical analyses when appropriate. Second, although our sample size is large for a PET study, it is relatively small for CT assessments, and this may have decreased the probability of identifying a correlation between CT and NS. Large populations are generally required to account for the maturational variability that occurs throughout late adolescence and early adulthood (i.e. the age group we examined) [65]. Further, even in large healthy populations, the association between externalizing traits (and personality traits in general) and brain morphometric features is generally subtle [3],[4]. Third, CT can be adjusted by total brain volume (TBV)/intracranial volume (ICV), grey matter volume or not at all. In the current study, we adjusted CT by TBV, following the recommendations of Zhou et al., 2014 [47]. By not including TBV as a covariate or control variable (in the partial correlations), the degrees of freedom were affected. We compensated for the potential influence of this decision on significance by setting stringent *p*-value thresholds for the CT analyses. In comparison, significant associations were not observed when using absolute CT values or CT values with TBV added as a covariate. It is plausible that the addition of TBV as a covariate decreased our power to detect effects, or that adjusted CT values are more sensitive in revealing a relation between striatal DA responses and cortical morphometry. Indeed, there is little consensus as to whether TBV/ICV should [66] or should not [4],[67],[68] be included as a covariate in CT analyses in the context of substance use research. More broadly, there is limited agreement as to how CT should be analyzed (i.e., absolute *vs*. corrected values). Thus, such methodological differences must be kept in mind when comparing and replicating future research. Further, we focused on TPQ-assessed NS, however, different externalizing measures may lead to different results, and should be investigated in future work. Finally, associations between NS, striatal DA release and CT may be nonlinear, and non-linear statistical approaches might reveal more complex associations. However, in our sample, exploratory non-linear correlations were not significant, and the current results strengthen the evidence for associations between striatal DA release, NS related impulsiveness and frontal cortical thickness in the largest known sample to date.

## Supporting information

S1 Table Excel table containing the dataset used in the analyses outlined in this manuscript. (XLSX)
